# Metallic Octahedral CoSe_2_ Threaded by N‐Doped Carbon Nanotubes: A Flexible Framework for High‐Performance Potassium‐Ion Batteries

**DOI:** 10.1002/advs.201800782

**Published:** 2018-08-07

**Authors:** Qiyao Yu, Bo Jiang, Jun Hu, Cheng‐Yen Lao, Yunzhi Gao, Peihao Li, Zhiwei Liu, Guoquan Suo, Donglin He, Wei (Alex) Wang, Geping Yin

**Affiliations:** ^1^ MIIT Key Laboratory of Critical Materials Technology for New Energy Conversion and Storage School of Chemistry and Chemical Engineering Harbin Institute of Technology Harbin 150001 China; ^2^ Department of Materials Science and Engineering NTNU Norwegian University of Science and Technology Trondheim 7491 Norway; ^3^ Institute for Advanced Materials and Technology University of Science and Technology Beijing Beijing 100083 China; ^4^ Department of Materials Science and Metallurgy University of Cambridge Cambridge CB3 0FS UK; ^5^ Department of Materials Science and Engineering Peking University Beijing 100871 China; ^6^ School of Materials Science and Engineering Shanxi University of Science and Technology Weiyang, Xi'an Shanxi 710021 China

**Keywords:** carbon nanotube frameworks, flexible electrodes, metallic CoSe_2_, octahedrons, potassium‐ion batteries

## Abstract

Due to the abundant and low‐cost K resources, the exploration of suitable materials for potassium‐ion batteries (KIBs) is advancing as a promising alternative to lithium‐ion batteries. However, the large‐sized and sluggish‐kinetic K ions cause poor battery behavior. This work reports a metallic octahedral CoSe_2_ threaded by N‐doped carbon nanotubes as a flexible framework for a high‐performance KIBs anode. The metallic property of CoSe_2_ together with the highly conductive N‐doped carbon nanotubes greatly accelerates the electron transfer and improves the rate performance. The carbon nanotube framework serves as a backbone to inhibit the agglomeration, anchor the active materials, and stabilize the integral structure. Every octahedral CoSe_2_ particle arranges along the carbon nanotubes in sequence, and the zigzag void space can accommodate the volume expansion during cycling, therefore boosting the cycling stability. Density functional theory is also employed to study the K‐ion intercalation/deintercalation process. This unique structure delivers a high capacity (253 mAh g^−1^ at 0.2 A g^−1^ over 100 cycles) and enhanced rate performance (173 mAh g^−1^ at 2.0 A g^−1^ over 600 cycles) as an advanced anode material for KIBs.

## 1 Introduction

Due to their high energy density and power density, lithium‐ion batteries (LIBs) have been widely used in portable electronics and electric vehicles.^1^, ^2^, ^3^, ^4^ However, with the rapid increase of commercial demands for large‐scale stationary application, the scarcity and uneven distribution of lithium source as well as the high cost are the main issues that need concern. Moreover, the safety is also a significant problem faced by LIBs caused by lithium dendrites.^5^, ^6^ Hence, developing new‐type energy storage devices with low cost and high safety is crucial as alternatives to LIBs, including sodium‐ion batteries (NIBs), magnesium‐ion batteries (MIBs), aluminum‐ion batteries (AIBs), and potassium‐ion batteries (KIBs).^6^, ^7^, ^8^, ^9^, ^10^, ^11^, ^12^, ^13^ Compared with the natural abundance of lithium (20 ppm) in the earth's crust, the storage of potassium (17 000 ppm) seems inexhaustible.^14^ Another virtue of KIBs is that K^+^/K showed more negative potential than Na^+^/Na, endowing them a higher full‐cell potential.

Generally, graphite is the common anode for KIBs with a maximum capacity of 279 mAh g^−1^, which has begun to attract the researchers' attention since three years.^10^, ^15^, ^16^ Due to the large interlayer spacing (3.4 Å) and high conductivity, the K ions can feasibly intercalate/deintercalate into/from the graphite layer.^17^ An obvious voltage plateau and relatively high capacity can be obtained, but the stability is not satisfied owing to the huge expansion and collapse of the crystal structure. Other carbonaceous materials like graphene, hard carbon, etc., also exhibit the K intercalation/deintercalation behavior, however, neither the capacity nor the cycle life is comparable to LIBs. Only a few K‐intercalated materials are studied, such as K_2_Ti_4_O_9_,^18^ K_2_Ti_8_O_17_,^19^ Sn_4_P_3_—C,^20^ Ti_3_C_2_ MXene,^21^ Sn—C,^22^ Sb—C,^23^ black P,^24^ etc., but the crystal structure is badly destroyed during cycling. Therefore, materials with high conductivity, stable structure, and high capacity are the ideal anodes for KIBs.

As a significant family of alkali‐ion intercalation materials, transition metal chalcogenides (named TMC) show high capacity, intrinsically enhanced safety, and high availability through conversion reaction.^25^, ^26^, ^27^, ^28^, ^29^ The drawbacks are also obvious, including poor electrical conductivity and huge volume expansion, causing poor rate performance and fast capacity decay.^30^, ^31^ To address the above issues, several approaches have been used such as seeking high‐conductivity TMC, designing TMC/carbon composites, fixing TMC onto conductive materials and synthesizing rationally engineered nanostructures.^32^, ^33^, ^34^ As a consequence, the above‐mentioned TMC composites are supposed to improve alkali‐ion storage behavior.

Herein, combining the aforementioned advantages, we for the first time design and construct a metallic octahedral CoSe_2_ threaded by N‐doped multiwall carbon nanotubes framework for high‐performance potassium storage. The bandgap of cobalt selenide is zero, indicating its conductor property, which is beneficial to rate performance. The N‐doped multiwall carbon nanotubes framework (NCNF) serves as multiple roles, including conductivity network, structural skeleton, restraining agglomeration, current collector, and even capacity contribution. More importantly, the zigzag void space among octahedral CoSe_2_ particles can accommodate the volume expansion after K‐ion intercalation and improve the structural stability. The first‐principles calculations are also used in this work to reveal the phase transition during charge/discharge process. This flexible electrode exhibits promising properties for practical application in energy storage system, especially for flexible and bendable devices.

## 2 Results and Discussions

**Figure 1:**
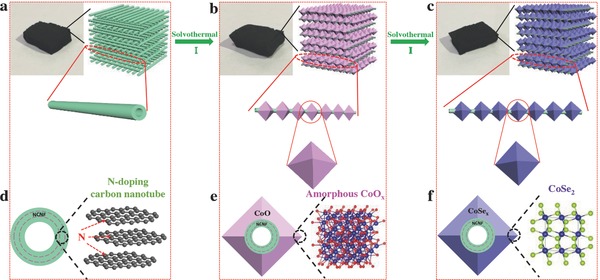
Fabrication of a) NCNF, b) NCNF@CoO*_x_*, and c) NCNF@CS. Cross section drawn and the corresponding structure of d) NCNF, e) NCNF@CoO*_x_*, and f) NCNF@CS.

The synthetic strategy of NCNF@CS is schematically illustrated in **Figure** **1**. The NCNF was first synthesized by a chemical vapor deposition (CVD) method^35^ using 1,2‐dichlorobenzene and ferrocene as carbon and catalyst sources (Figure 1a). Then CoCl_2_•6H_2_O, NH_4_F, and urea were mixed together at the molecular level to achieve homogenous phase distribution in aqueous solution. The NCNF was dropped into the above solution and after a typical solvothermal synthesis, the NCNF@CoO*_x_*‐*n* (*n* is the reaction hours) core–shell is obtained (Figure 1b). In this process, each N‐doped carbon nanotube (CNT) in NCNF is covered by amorphous octahedral CoO*_x_*, forming NCNF@CoO*_x_* skeleton frame. The subsequent step is the selenation process by solvothermal method using selenous acid and hydrazine hydrate as selenium source and reductant. Finally, the octahedral cobalt selenide threaded by N‐doped carbon nanotubes framework NCNF@CS‐*n* is obtained (Figure 1c). In addition, the cross‐section drawn and the corresponding structure are also illustrated to give a detailed observation (Figure 1d–f).

**Figure 2:**
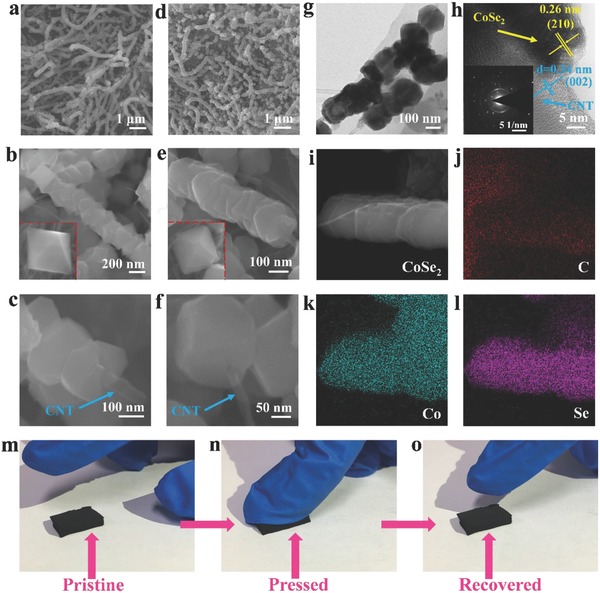
SEM images of a–c) NCNF@CoO*_x_*‐6h and d–f) NCNF@CS‐6h. g) TEM and h) HRTEM images of NCNF@CS‐6h. The insets of (b) and (e) inside red dashed box are the magnified corresponding particles. The inset of (h) is corresponding SAED pattern. i) HAADF‐STEM image and EDS mappings of j) C, k) Co, and l) Se of NCNF@CS‐6h. Photos of flexibility test using NCNF@CS‐6h: m) pristine, n) under pressure, and o) after pressure released.

The scanning electron microscopy (SEM) and transmission electron microscopy (TEM) images show that the NCNF consists of a large number of multiwall carbon nanotubes with a diameter of tens of nanometers and a length of several micrometers (Figures S1 and S2, Supporting Information). The X‐ray diffraction (XRD) pattern of the NCNF along with the Raman spectrum exhibits typical CNT characteristic (Figure S3, Supporting Information). The SEM images of the NCNF@CoO*_x_* with different magnifications show that the octahedral CoO*_x_* particles are threaded by every N‐doped carbon nanotube (NCNT) and lined up in a row (**Figure** **2**a–c). Inside red dashed box is the magnified representative CoO*_x_* particles, showing a clear octahedral structure (inset of Figure 2b). Under a larger magnification, it can be seen clearly that the CNT pierces the CoO*_x_* octahedron and one end of the CNT comes out (Figure 2c). The as‐prepared NCNF@CS‐6h has inherited the octahedral shape of the parent NCNF@CoO*_x_*‐6h without collapse (Figure 2d–f). Notably, every octahedral CoSe_2_ particle arranges along the carbon nanotubes in sequence, leaving zigzag void space among particles. This feature has the ability to accommodate the volume expansion after K‐ion intercalation and improve its structural stability. The TEM image (Figure 2g) as well as the SEM images of the NCNF@CS‐6h demonstrate that the diameter of single octahedral cobalt selenide particle is in the range of 150–200 nm. The high‐resolution TEM (HRTEM) image and the corresponding selected area electron diffraction (SAED) pattern show the polycrystalline characteristic of cobalt selenide (Figure 2h). For comparison, the SEM images of NCNF@CS‐3h and NCNF@CS‐12h are also displayed (Figure S4, Supporting Information). The cobalt selenide in NCNF@CS‐3h is cracked and easy to fall off with much lower loading content (Table S1, Supporting Information), while the cobalt selenide particles in NCNF@CS‐12h are densely arranged. The weight ratios of cobalt selenide to NCNTs in NCNF@CS‐3h, NCNF@CS‐6h, and NCNF@CS‐12h are calculated to be 1.55, 2.38, and 4.16, respectively. The high‐angle annular dark‐field scanning transmission electron microscopy (HAADF‐STEM) image and energy‐dispersive X‐ray spectra (EDS) elemental mapping results of cobalt selenide clearly reveal that the elements of Co, Se, and C are uniformly distributed throughout the NCNF@CS‐6h (Figure 2i–l), inheriting the uniform distribution of NCNF@CoO*_x_*‐6h (Figure S5, Supporting Information). The EDS spectra of both NCNF@CoO*_x_*‐6h and NCNF@CS‐6h are also shown in Figures S6 and S7 (Supporting Information). To testify the flexibility, we pressed the as‐prepared NCNF@CS‐6h and then released the pressure (Figure 2m–o). As expected, the NCNF@CS‐6h fully recovered to the original volume. The flexible and tensile testing was also carried out in Figure S8 (Supporting Information), indicating its high flexibility, which can be applied in flexible battery.

**Figure 3:**
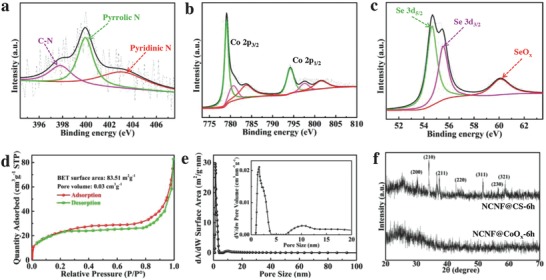
a) XPS spectra of N 1s in NCNF. XPS spectra of b) Co 2p and c) Se 3d in NCNF@CS‐6h. d) Nitrogen adsorption–desorption isotherm and e) pore size distribution of NCNF@CS‐6h. The inset of (e) is the partial enlarged detail. f) XRD patterns of the NCNF@CS‐6h and NCNF@CoO*_x_*‐6h.

The X‐ray photoelectron spectroscopy (XPS) was carried out to investigate the chemical composition and the surface electronic state of the as‐prepared NCNF@CS‐6h (**Figure** **3**a–c). Using deconvolution method, the N 1s spectrum was fitted by the assumption of three species: C—N (397.8 eV), pyrrole N (399.9 eV), and pyridinic N (403.2 eV) (Figure 3a).^36^ The HAADF‐STEM image, the EDS mappings, and the EDS spectra of the NCNF also prove the successful doping of N in CNT (Figures S9 and S10, Supporting Information). The N‐doped carbon can provide a large number of active sites and increase the electronic conductivity, enhancing the capacity and rate capability.^37^ The Co 2p_3/2_ peak can be deconvoluted into three chemical states at the binding energies of 779.0, 780.7, and 783.8 eV, which are assigned to the Co—Se, Co—O (a thin amorphous oxide layer on the surface) binding structures, and the shakeup satellite peak (Figure 3b).^38^, ^39^ The binding energies of Se 3d_3/2_ and Se 3d_5/2_ at 55.5 and 54.6 eV, respectively, also coincide with CoSe_2_.^38^ It is important to highlight that the d‐electron configuration of metal ions has an important impact on the physical properties the metal dichalcogenides. The 3d bands of the metal atoms can further split into the sub‐bands (t_2g_ and e_g_) by a crystalline field. Thus, the Co 3d electrons of CoSe_2_ adopt a low‐spin configuration in the form of t_2g_^6^e_g1_, making it a metallic conductor.^40^ The metallic property benefits efficient transport of electrons through the electrode of KIBs, which is also desired for high‐rate performance.

The N_2_ adsorption/desorption isotherm profiles of the NCNF@CS‐6h are shown in Figure 3d. The isotherms are obtained with a large Brunauer—Emmett–Teller (BET) surface area of 83.5 m^2^ g^−1^. A very large proportion of the pore size is centered at 1.5 nm, and meanwhile a small portion of pores peaked at 10.2 nm are observed, representing the existence of the micropores and mesopores (Figure 3e). It should be pointed out that the as‐synthesized NCNF@CoO*_x_*‐6h is amorphous, while after the selenation process, the crystal structure (JCPDS No. 65‐3327) is obtained (Figure 3f). The HRTEM and the corresponding SAED pattern (Figure S11, Supporting Information) further prove the amorphous nature of CoO*_x_*. The Raman spectrum of the synthesized NCNF@CS‐6h shows the existence of carbon and cobalt selenide (Figures S12 and S13, Supporting Information). It can be clearly seen that the the D‐band (assigned to A_1g_ vibration mode of sp^2^ carbon rings caused by defects) is more intensive than the G‐band (assigned to E_2g_ vibration mode of sp^2^ carbon atoms) with an *I*_D_/*I*_G_ value of 1.12, indicating its low degree of graphitization.

**Figure 4:**
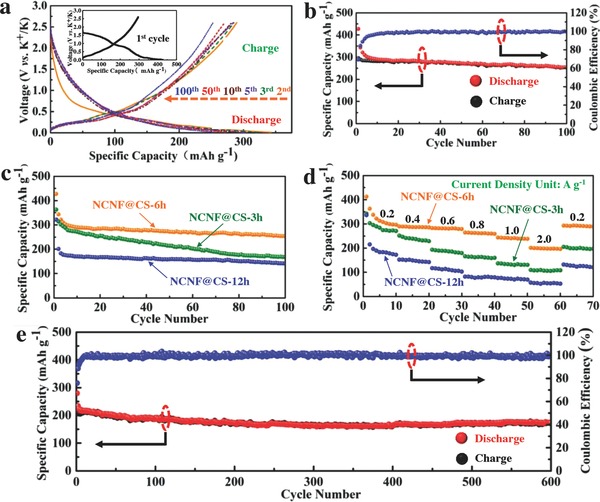
a) The 2nd, 3rd, 5th, 10th, 50th, and 100th charge/discharge profiles of NCNF@CS‐6h at 0.2 A g^−1^ for KIBs (inset is the charge/discharge profiles of the first cycle). b) Charge/discharge capacity and Coulombic efficiency of NCNF@CS‐6h at 0.2 A g^−1^. Comparison of c) cycling stability and d) rate performance under different current densities of the as‐prepared three NCNF@CS samples. e) Long‐term cycling stability and Coulombic efficiency at a high current density of 2.0 A g^−1^ over 600 cycles.

The galvanostatic charge/discharge curves of NCNF@CS‐6h are shown in **Figure** **4**a. To get a clear view, the initial charge/discharge curves are separated out (inset of Figure 4a). The charge/discharge capacities for the initial cycle are 296 and 427 mAh g^−1^, respectively, with a Coulombic efficiency of 69.3%. The extremely high capacity and the remarkable capacity loss between the initial and second cycle are mainly attributed to some irreversible processes like the formation of the solid electrolyte interphase (SEI) layer on the NCNF@CS‐6h surface,^41^ agreeing well with the results of cyclic voltammetry (CV) curves (Figure S14, Supporting Information). These irreversible processes caused by the formation of SEI and other side reactions in initial ten cycles can also result in the low Coulombic efficiency. In the subsequent cycles, the SEI layers become stable and robust, which can protect the active materials from further reacting with the electrolyte.^42^ Therefore, the Coulombic efficiency is gradually increased. During cycling, the majority of its voltage is higher than the plating voltage of potassium metal (0.01 V), therefore lowering the risk of short‐circuiting due to the dendrite growth. Figure S15 (Supporting Information) shows the SEM images of K metal surface after different cycles. When cycled at 0.2 A g^−1^ for 100 cycles, although the K metal cracks into pieces, no obvious K dendrites growth can be observed.

The cycling stability and Coulombic efficiency of the NCNF@CS‐6h at 0.2 A g^−1^ over 100 cycles are shown in Figure 4b. In the initial several cycles, although the capacity is a bit higher, yet the capacity fading is severer, which may be due to the side reaction, as mentioned in Figure 4a. Nevertheless, after the first five cycles, high capacity retention and Coulombic efficiency are obtained, demonstrating a high reversibility during cycling. A capacity of 253 mAh g^−1^ after 100 cycles with the capacity retention of ≈85.3% is achieved, indicating good cycling stability. To gain a better understanding, the electrochemical performance of the individual NCNF is also measured (Figures S16 and S17, Supporting Information). It shows quite good capacity and cycling stability as carbonaceous material anode, indicating one of its multiroles, the capacity contribution. In spite of this, the capacity is still much lower than the NCNF@CS‐6h. It must also be mentioned that the specific capacity of the NCNF@CS is calculated using the whole NCNF@CS mass as the weight of the active materials. For NCNF@CS‐6h, the weight ratio of cobalt selenide:NCNT is 2.38, and the real specific capacity of CoSe_2_ is calculated to be 289 mAh g^−1^ (corresponding to 253 mAh g^−1^ of the whole NCNF@CS mass after 100 cycles). Therefore, as the composite of the NCNF and cobalt selenide, the NCNF@CS‐6h not only inherits the conductive NCNT backbones but also possesses higher capacity.

The comparison of the cycling stability for the as‐prepared three NCNF@CS samples was performed at 0.2 A g^−1^ for KIBs (Figure 4c). Apparently, the NCNF@CS‐6h exhibits much better capacity and cycling stability than the other two samples. Similarly, the NCNF@CS‐6h also displays the best rate performance under different current densities from 0.2 to 2.0 A g^−1^ (Figure 4d). Reversible capacities of 297, 286, 279, 259, 238, and 196 mAh g^−1^ are observed after increasing the charge/discharge current densities to 0.2, 0.4, 0.6, 0.8, 1.0, and 2.0 A g^−1^. Remarkably, after 60 cycles under different current densities, the capacity can be restored to 293 mAh g^−1^ when the current density returns to 0.2 A g^−1^, indicating very excellent rate performance and reversibility. The corresponding Coulombic efficiencies are also shown in Figure S18 (Supporting Information). The following reasons can explain the differences in performance. The cobalt selenide in NCNF@CS‐3h is cracked and easy to fall off, leading to a fast capacity fading. Additionally, its much lower loading content is another factor for the unsatisfactory capacity. The NCNF@CS‐12h with densely arranged octahedral cobalt selenide particles is too tight to have full access to the electrolyte and has poor self‐volume‐accommodation ability. Distinctively, the octahedral cobalt selenide particles are distributed uniformly through the NCNF@CS‐6h and threaded and anchored firmly by every NCNT, resulting in better electrochemical activity.

To highlight the superiority of the unique NCNF@CS‐6h anode material, long‐term cycling stability of NCNF@CS‐6h at a very high current density of 2.0 A g^−1^ over 600 cycles is presented (Figure 4e). A reversible capacity of 173 mAh g^−1^ is achieved over such a long‐time charge/discharge process with a capacity fading of only 0.03% cycle^−1^. Notably, its Coulombic efficiency nearly comes up to 100%, indicating an excellent reversibility during cycling. It should be noted that the Coulombic efficiency of the electrode in high current density is higher than that in low current density (Figure 4b,e), which can be explained as follows. When discharged at a low current density, the depth of discharge (DOD) is high, which can cause a higher degree of damage to the electrode than that at a high current density. Therefore, a low Coulombic efficiency is obtained.^43^ The electrochemical performance of the recent reported anode materials for KIBs is shown for comparison (Table S2, Supporting Information). At the same time, the morphology after 600 cycles is observed by SEM, causing minor damage to the NCNF@CS‐6h (Figure S19, Supporting Information). Furthermore, the optical photographs of the electrode of the original and after 200 cycles (Figure S20, Supporting Information) show that the electrode structure and the thickness remain unchanged during cycling, indicating high structural stability.

To evaluate the electrode kinetics, the electrochemical impedance spectroscopy (EIS) was performed in a frequency range from 10^5^ to 0.01 Hz. The Nyquist plots of the NCNF@CS‐6h‐based coin cell are shown in Figure S21a (Supporting Information). Each impedance spectrum consists of two parts: the depressed semicircle at high frequency corresponds to the charge‐transfer impedance and the slope line at low frequency corresponds to the semi‐infinite diffusion of K ions into the electrolyte/electrode interface. Obviously, the 10th and 100th curves almost overlap, demonstrating good conductivity. The Nyquist plots were also fitted by the equivalent circuit model (Figure S21b, Supporting Information). *R*_s_ is the internal resistance of the coin cell and *C* is the capacitance. *R*_ct_ represents the charge‐transfer resistance attributed to the surface impedance and CPE is the constant phase‐angle element corresponding to the double‐layer capacitance. *W* represents the Warburg impedance that corresponds to the K diffusion process.

The diffusion coefficient of the potassium ions (*D*_K_) was also calculated by the following equations^44^, ^45^

(1) $$D_{K}=0.5R^{2}T^{2}/A^{2}n^{4}F^{4}C^{2}\sigma^{2}$$

In this equation, *R* is 8.314 J mol^−1^ K^−1^ (gas constant), *T* is 298 K (absolute temperature), *A* is 0.78 cm^2^ (the active surface area of the electrode/electrolyte interface), *n* is 1 (the number of the transferred electrons), *F* is 96 500 C mol^−1^ (the Faraday constant), *C* is ≈0.8 × 10^−3^ mol cm^−3^ (the bulk concentration), and σ = d*Z*′/dω^−1/2^ (Warburg coefficient, in low‐frequency region). According to the linear relationship between *Z*′ and ω^−1/2^ (Figure S21c, Supporting Information), the Warburg coefficient σ is 209.25 Ω s^1/2^. Hence, the diffusion coefficient *D*_K_ is calculated to be 4.34 × 10^−10^ cm^2^ s^−1^, comparable to that of LIBs anodes.^46^ The corresponding impedance parameters and Warburg and diffusion coefficient of the first curve are listed in Table S3 (Supporting Information). These results further demonstrate the high‐rate performance of NCNF@CS‐6h for KIBs.

To evaluate the capacity contributed by the capacitive behavior, we test the CV curves at stepped scan rates from 0.1 to 1.0 mV s^−1^ in a voltage range from 0.01 to 2.5 V (Figure S22, Supporting Information). Based on the previous reports,^47^, ^48^ the peak current (*i*) and the scan rate (υ) abide by the relationship of *i = av^b^*. Meanwhile, the *b* value can be obtained by the slope of the log (*i*) versus log(υ) plot. When the *b* value is close to 0.5, the electrochemical behavior is dominated by the ionic diffusion process, while a *b* value close to 1.0 indicates a total capacitive process.^47^, ^48^ Specifically, the capacitive contribution ratio under different scan rates can be quantified through the equation of *i = k*_1_ *v + k*_2_ *v* ^1/2^, where *k*_1_ *v* and *k*_2_ *v* ^1/2^ represent the contribution of capacitance and ionic diffusion, respectively. Under different scan rates, all the *b* values are below 0.5, indicating the charge storage behavior is dominated by the ionic diffusion process, but the capacity contributed by the capacitive behavior cannot be ignorable (Figure S22b–d, Supporting Information). As the scanning rate increases, the capacitive charge contribution becomes higher and finally reaches the maximum value of 42.2% at a high scan rate of 1.0 mV s^−1^. The nonignorable capacitive contribution is in favor of the capacity and rate performance of NCNF@CS‐6h.

The reasons for the high‐performance KIBs are as follows: (i) Owing to its metallic property, the transport of electrons in CoSe_2_ is faster than other insulating anode materials (metal oxides, metal phosphides, potassium titanates, etc.), in favor of the rate performance. (ii) The NCNF plays multiple roles in this work. Each carbon nanotube can firmly anchor the octahedral CoSe_2_ particles by piercing their center and prevent the agglomeration, obtaining a uniformly distributed CoSe_2_ on NCNF. This unique structure can accommodate the volume expansion and achieve long‐term cycling stability during charge/discharge process. The skeleton frame serves as a conductive network, meanwhile every CoSe_2_ particle is connected in series through carbon nanotubes. Therefore, the electrons can easily transfer to each CoSe_2_ particle from external circuit or in an opposite direction (Figure S23, Supporting Information), improving the rate capability. (iii) As one of the transition metal chalcogenides, a high theoretical capacity is predictable. Through the efforts in this work, its high capacity can be maintained even at high current densities and in a long period. (iv) The nonignorable capacitive contribution is another significant factor for the high capacity and rate performance.

**Figure 5:**
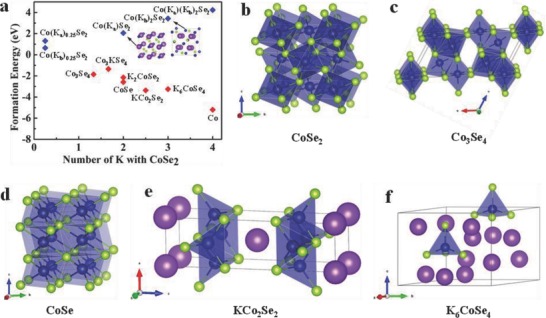
a) The relationship between the formation energy and the number of K. The red dots show the formation energy to stable compounds, and the blue dots show the formation energy of insertion K. b–f) Schematic molecular structures using in this work.

In order to better understand the intercalation process, the first‐principle calculations were employed to theoretically investigate the K ions into the CoSe_2_ crystal structure. The calculated formation energies (*E*_f_) with respect to the number of K ions react with CoSe_2_ and schematic molecular structures are shown in **Figure** **5**. The structure information and total energy of different compositions are shown in Table S4 (Supporting Information).

In the K‐intercalation reaction, *K*_a_ (0.5 0.5 0.5) and *K*_b_ (0.25 0.25 0.25) means the two kinds of equivalent position in CoSe_2_

(2) $${\mathrm{CoSe}}_{2}+K^{+}+e^{-}\to \mathrm{Co}\left(K_{a}\right){\mathrm{Se}}_{2},E_{f}=2.06\;\mathrm{eV}$$

(3) $${\mathrm{CoSe}}_{2}+2K^{+}+2e^{-}\to \mathrm{Co}{\left(K_{b}\right)}_{2}{\mathrm{Se}}_{2},E_{f}=3.43\mathrm{eV}$$

(4) $${\mathrm{CoSe}}_{2}+3K^{+}+2e^{-}\to \mathrm{Co}\left(K_{a}\right){\left(K_{b}\right)}_{2}{\mathrm{Se}}_{2},E_{f}=4.24\mathrm{eV}$$

(5) $${\mathrm{CoSe}}_{2}+4K^{+}+4e^{-}\to {\mathrm{Co+2K}}_{2}\mathrm{Se},E_{f}=-5.20\mathrm{eV}$$

(6) $${\mathrm{1/3Co}}_{3}{\mathrm{Se}}_{4}+1/3K^{+}+1/3e^{-}\to 1/3{\mathrm{Co}}_{3}{\mathrm{KSe}}_{4},\;E_{f}=-0.39\mathrm{eV}$$

The formation energies of reactions (2)–(4) are positive, hence suggesting that the three insertion reactions are not favorable during cycling. However, the replacement reaction from CoSe_2_ to Co and K_2_Se can happen spontaneously. It can be seen that reaction (5) exhibits negative formation energy, suggesting that the insertion reactions for K ions into Co_3_Se_4_ are energetically favorable. Careful examination of the structures indicates the potassium atom is too large to penetrate the CoSe_2_ crystal structure lattice, while the existence of interspace of nonstoichiometric selenides CoSe_1−_ *_x_*(Co_3_Se_4_) makes possible. These phenomena are quite different from the intercalation of Li ions in CoSe_2_.^49^ By combining Figure 5 and Table S4 (Supporting Information), the actual reaction process from CoSe_2_ to Co and K_2_Se can be explained as follows

(7) $${\mathrm{CoSe}}_{2}+4/3K^{+}+4/3e^{-}\to 1/3{\mathrm{Co}}_{3}{\mathrm{Se}}_{4}+2/3K_{2}\mathrm{Se}$$

(8) $${\mathrm{Co}}_{3}{\mathrm{Se}}_{4}+K^{+}+e^{-}\to {\mathrm{Co}}_{3}{\mathrm{KSe}}_{4}$$

(9) $${\mathrm{Co}}_{3}{\mathrm{KSe}}_{4}+K+e^{-}\to 3{\mathrm{CoSe+K}}_{2}\mathrm{Se}$$

(10) $${\mathrm{CoSe+2K}}^{+}+2e^{-}\to {\mathrm{Co+K}}_{2}\mathrm{Se}$$

The last thing worth mentioning is that the bandgap of different compositions during intercalation process is zero except the final formed K_2_Se (Table S5, Supporting Information), beneficial to the electron conduction throughout the whole intercalation process. The corresponding bandgap structure and density of states are also shown in Figures S24–S29 (Supporting Information).

## 3 Conclusions

In summary, we have synthesized a metallic octahedral cobalt selenide threaded by N‐doped carbon nanotubes framework for the first time as an advanced anode material for KIBs. The NCNF plays multiple roles in this work, including conductivity network, structural skeleton, restraining agglomeration, current collector, and even capacity contribution. The metallic CoSe_2_ itself can provide fast electrons transport and high theoretical capacity, which is also in favor of high‐performance KIBs. In addition, every octahedral CoSe_2_ particle arranges along the carbon nanotubes in sequence, leaving zigzag void space among particles, which can accommodate the volume expansion and improve the structural stability during cycling. It can deliver a high capacity of 253 mAh g^−1^ after 100 cycles with the capacity retention of ≈85.3%. Even at a very high current density of 2.0 A g^−1^ over 600 cycles, the capacity can still maintain 173 mAh g^−1^, with a capacity fading of only 0.03% cycle^−1^, indicating high rate performance and excellent cycling stability.

## 4 Experimental Section

*Chemicals*: CoCl_2_•6H_2_O (AR, Aladdin), NH_4_F (AR, Aladdin), urea (AR, Aladdin), anhydrous ethanol (99.5%, Beijing Chemical Reagent Factory), hydrazine hydrate (80%, Aladdin), and H_2_SeO_3_ (98%, Aldrich).

*Synthesis of NCNF@CoO_x_*: All the reagents were used after purchase and without purification. The NCNF was first synthesized by a CVD method according to previous report, using 1,2‐dichlorobenzene and ferrocene as carbon and catalyst sources.^35^ The NCNF@CoO*_x_* was obtained by a hydrothermal method. In a typical procedure, 0.2 g CoCl_2_•6H_2_O, 0.06 g NH_4_F, and 0.48 g urea were dissolved in 40 mL ultrapure water at room temperature to form a homogeneous precursor solution under magnetic stirring. Then the NCNF (≈8 mg) was immersed into the anhydrous ethanol for only several seconds to improve the hydrophilic property before it was added into the above precursor. The mixture was then placed into a Teflon‐lined stainless‐steel autoclave and stirred for 20 min. The autoclave was heated at 180 °C for 3, 6, and 12 h in an oven and then cooled to room temperature naturally. The obtained NCNF@CoO*_x_* was washed with ultrapure water and ethanol for several times and dried at 80 °C overnight under ambient atmosphere.

*Synthesis of NCNF@CS*: The selenation process was carried out by a hydrothermal method. Typically, the obtained NCNF@CoO*_x_* (3, 6, and 12 h) and 0.258 g H_2_SeO_3_ were dissolved into 42 mL ultrapure water and hydrazine hydrate (80%) with a volume ratio of 1:1. The above three mixtures were placed into the Teflon‐lined stainless‐steel autoclaves and stirred for 20 min. The autoclaves were heated at 120 °C for 10 h in an oven and then cooled to room temperature naturally. The final products were collected by dispersing them in ultrapure water and ethanol for 30 min, followed by evaporation and vacuum drying at 60 °C for 6 h.

*Characterization*: The compositions of the synthesized samples were studied by XRD by a Rigaku D/max‐RB12 X‐ray diffractometer with Cu Kα radiation. The morphology and composition of the products were characterized by SEM (JEOL, JSM‐7001F). TEM and HRTEM images were observed by a transmission electron microscopy (JEOL, JEM‐2010). STEM analyses and EDS were carried out using a Hitachi HD2700C (200 kV). XPS were tested with an ESCALAB 250 spectrometer (Perkin Elmer) to characterize the surface chemistry. The Raman spectra were tested through a microscopic confocal Raman spectrometer (Renishaw RM2000) with a wavelength of 514.5 nm at room temperature. The surface area was measured according to the BET (Micromeritics Instrument Corporation 3Flex) method.

*Electrochemical Characterization*: Electrochemical measurements were carried out using the CR2032‐type coin cells. The working electrode was the as‐synthesized NCNF@CS (or NCNF), and the potassium metal worked as both the counter and reference electrodes. The loading of electrodes placed into the coin cells was around 1.5–2.0 mg cm^−2^. A nonaqueous solution of 0.8 m KPF_6_ in a 1:1 (v/v) mixture of ethylene carbonate (EC) and diethyl carbonate (DEC) was used as the electrolyte. The glass fiber (GF/D) from Whatman was used as the separators. The coin cells were assembled and disassembled in an argon‐filled glove box. The coin cells were galvanostatically charged and discharged at different current densities within a voltage range of 0.01–2.5 V, using LAND‐CT2011A battery‐testing instrument under room temperature. CVs scanned in a voltage window of 0.01–2.5 V at 0.1 mV s^−1^ were measured by an electrochemistry workstation (CHI618D).

*Computational Section*: The first principle calculations were performed with the Vienna Ab initio Simulation Package (VASP)^50^, ^51^ using the PBEsol functional.^52^, ^53^ The standard PBE PAW potentials K (3s^2^3p^6^4s), Co (3d^7^4s^2^), and Se (4s^2^4p^4^) potentials supplied with VASP were used and plane waves were expanded up to a cutoff energy of 500 eV. The SCF convergence energy was set to 1 × 10^−6^ eV the Hellman–Feynman forces on the ions were relaxed until they were below 0.001 eV Å^−1^ for all calculations. Brillouin zone interagrion was done on a 5 × 5 × 5 mesh for CoSe_2_ with 12 atoms, and the same dense mesh for other compounds. The optimization terminates when all of these criteria are satisfied. The choice of these computational parameters ensures good convergence in present studies.

Previous X‐ray study^54^ shows that the cobalt selenides have revealed three intermediate solid phase, β‐phase, Co_9_Se_8_, γ‐phase, CoSe‐Co_3_Se_4_,^55^ and δ‐phase, CoSe_2_. The CoSe_2_ and Co_3_Se_4_ were chosen as the initial structure for potassium doped/replacement reaction. Compounds based on K with CoSe*_x_* (K_2_CoSe_2_, KCo_2_Se_2_,^56^ K_6_CoSe_4_ 57) will also be considered as reaction products in the calculation process.

As potassium reacted with the CoSe_2_ and Co_3_Se_4_, refer to the following reaction

(11) $${\mathrm{CoSe}}_{2}+xK\to K_{\left(x-2z\right)}{\mathrm{Co}}_{\left(1-y\right)}{\mathrm{Se}}_{\left(2-z\right)}+y\mathrm{Co}+zK_{2}\mathrm{Se}$$

(12) $${\mathrm{1/3Co}}_{3}{\mathrm{Se}}_{4}+xK\to K_{\left(x-2z\right)}{\mathrm{Co}}_{\left(1-y\right)}{\mathrm{Se}}_{\left(\mathrm{4/3}-z\right)}+y\mathrm{Co+}zK_{2}\mathrm{Se}$$

When *y* and *z* equal to zero, which means the insertion K reaction

(13) $${\mathrm{CoSe}}_{2}+xK\to {\mathrm{CoK}}_{x}{\mathrm{Se}}_{2}$$

(14) $$\begin{array}{c} {\mathrm{1/3Co}}_{3}{\mathrm{Se}}_{4}+xK\to {\mathrm{1/3Co}}_{3}K_{3x}{\mathrm{Se}}_{4} \end{array}$$

So the formation energy for reactions (1)–(4) is defined as

(15) $$\begin{array}{l} E_{f}\left(M\right)=\;E_{\mathrm{tot}}\left(K_{\left(x-2z\right)}{\mathrm{Co}}_{\left(1-y\right)}{\mathrm{Se}}_{\left(2-z\right)}\right)+yE_{\mathrm{tot}}\left(\mathrm{Co}\right)+zE_{\mathrm{tot}}\left(K_{2}\mathrm{Se}\right)\;\\ \;\;\;\;\;\;\;\;\;\;\;\;\;\;\;\;\;-xE_{\mathrm{tot}}\left(K\right)\;-\;E_{\mathrm{tot}}\left({\mathrm{CoSe}}_{2}\right) \end{array}$$

(16) $$\begin{array}{l} E_{f}\left(M\right)=E_{\mathrm{tot}}\left(K_{\left(x-2z\right)}{\mathrm{Co}}_{\left(1-y\right)}{\mathrm{Se}}_{\left(\mathrm{4/3}-z\right)}\right)+yE_{\mathrm{tot}}\left(\mathrm{Co}\right)+zE_{\mathrm{tot}}\left(K_{2}\mathrm{Se}\right)\;\\ \;\;\;\;\;\;\;\;\;\;\;\;\;\;\;\;\;-\;xE_{\mathrm{tot}}\left(K\right)\;-\;E_{\mathrm{tot}}\left({\mathrm{1/3Co}}_{3}{\mathrm{Se}}_{4}\right) \end{array}$$

(17) $$E_{f}\left(M\right)=E_{\mathrm{tot}}\left({\mathrm{CoK}}_{x}{\mathrm{Se}}_{2}\right)-xE_{\mathrm{tot}}\left(K\right)-\;E_{\mathrm{tot}}\left({\mathrm{CoSe}}_{2}\right)$$

(18) $$E_{f}\left(M\right)=E_{\mathrm{tot}}\left({\mathrm{1/3Co}}_{3}K_{3x}{\mathrm{Se}}_{4}\right)-xE_{\mathrm{tot}}\left(K\right)-E_{\mathrm{tot}}\left({\mathrm{1/3Co}}_{3}{\mathrm{Se}}_{4}\right)$$

Here, *E*_tot_ is the total energy of corresponding compounds, and *E*_tot_(K) and *E*_tot_(Co) are the total energy of bulk K and Co.

## Conflict of Interest

The authors declare no conflict of interest.

## Supporting information

Supplementary
