# Supplementary material for: Metallic Octahedral CoSe2 Threaded by N‐Doped Carbon Nanotubes: A Flexible Framework for High‐Performance Potassium‐Ion Batteries
Source: Adv Sci (Weinh). 2018 Aug 7;5(10):1800782. doi: 10.1002/advs.201800782 (PMC6193164; doi:10.1002/advs.201800782)
Supplement: Supplementary file 1 — Supplementary [file ADVS-5-1800782-s001.pdf]

Copyright WILEY-VCH Verlag GmbH & Co. KGaA, 69469 Weinheim, Germany, 2018.

ADVANCED SCIENCE
Open Access

Supporting Information

for *Adv. Sci.*, DOI: 10.1002/advs.201800782

Metallic Octahedral $CoSe_2$ Threaded by N-Doped Carbon Nanotubes: A Flexible Framework for High-Performance Potassium-Ion Batteries

Qiyao Yu, Bo Jiang, Jun Hu, Cheng-Yen Lao, Yunzhi Gao, Peihao Li, Zhiwei Liu, Guoquan Suo,* Donglin He, Wei (Alex) Wang,* and Geping Yin*

Supporting Information

Metallic Octahedral $CoSe_2$ Threaded by N-doped Carbon Nanotubes: A Flexible Framework for High-Performance Potassium-Ion Batteries

Qiyao Yu, Bo Jiang, Jun Hu, Chengyen Lao, Yunzhi Gao,* Peihao Li, Zhiwei Liu, Guoquan Suo,* Donglin He, Wei Wang,* GepingYin

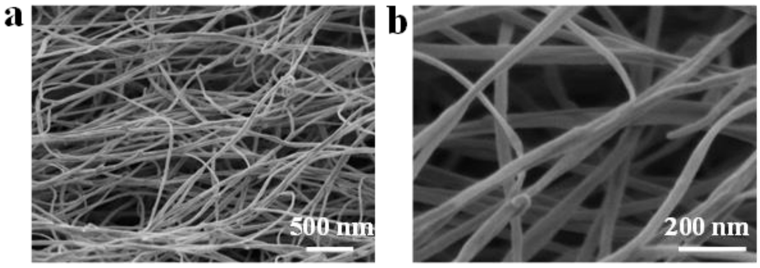

Figure S1. SEM images of NCNF.

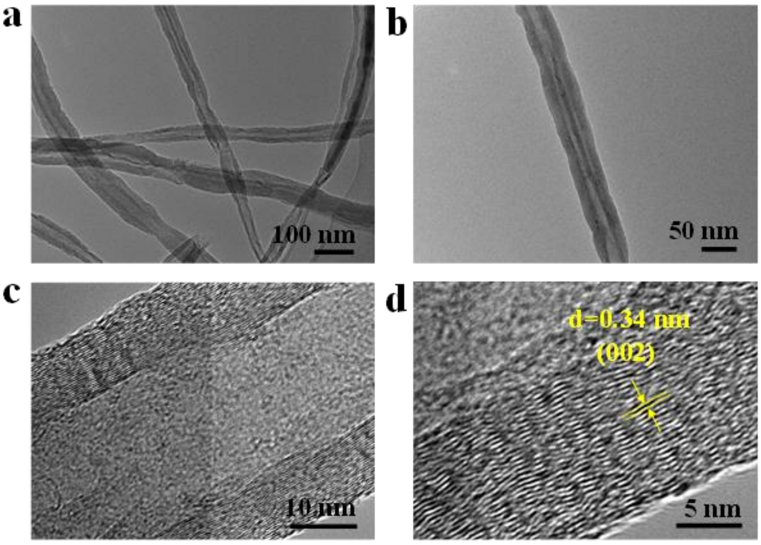

Figure S2. (a,b) TEM and (c,d) HRTEM images of NCNF.

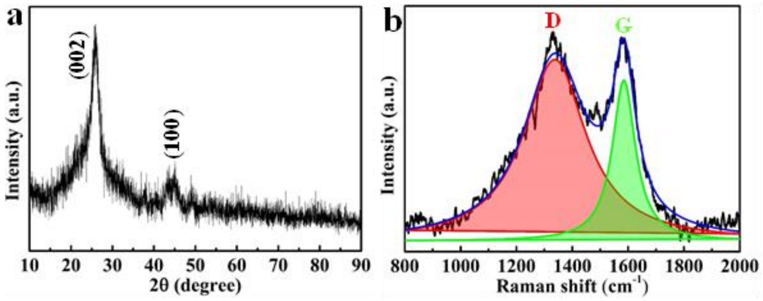

Figure S3. (a) XRD pattern and (b) Raman spectrum of the NCNF.

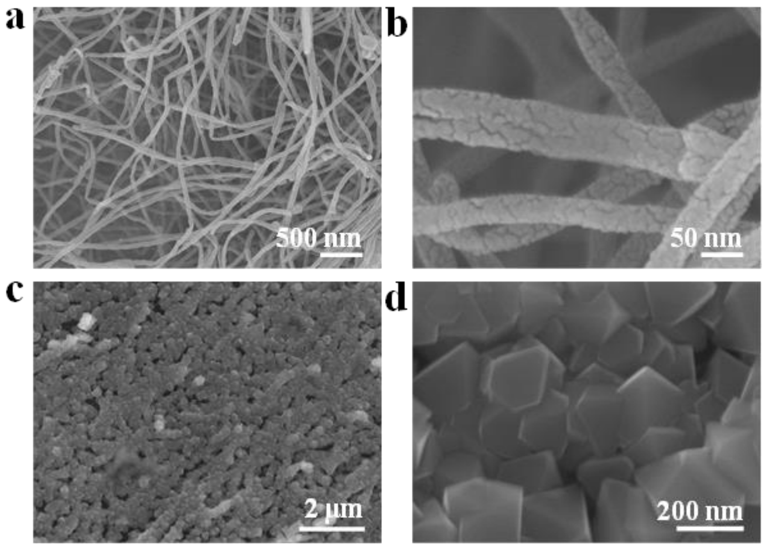

Figure S4. SEM images of (a,b) NCNF@CS-3h and (c,d) NCNF@CS-12h.

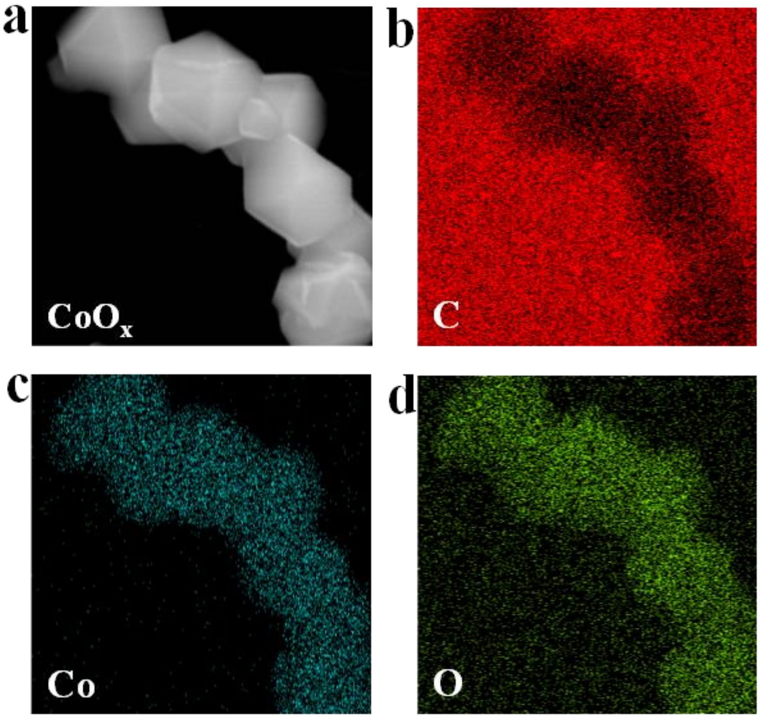

Figure S5. (a) HAADF-STEM image and EDS mappings of (b) C, (c) Co and (d) O of NCNF@CoO_x-6h.

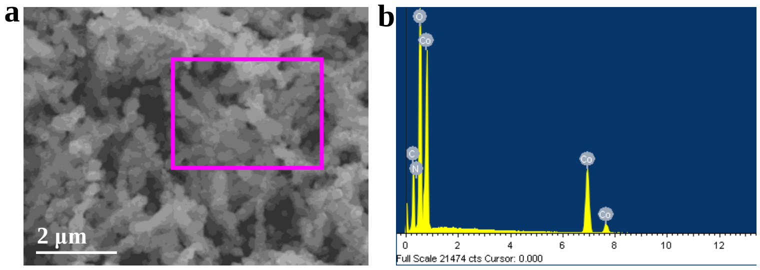

Figure S6. (a) SEM image and EDS spectra of NCNF@CoO_x-6h.

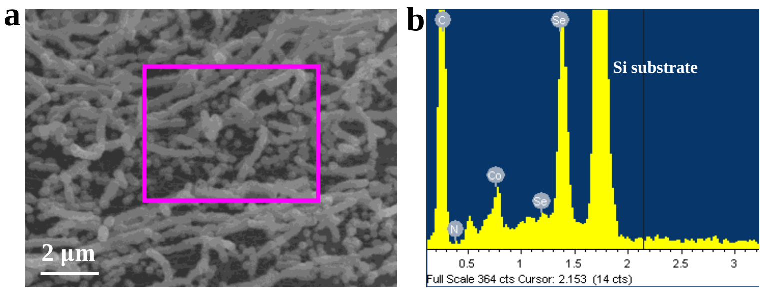

Figure S7. (a) SEM image and EDS spectra of NCNF@CS-6h.

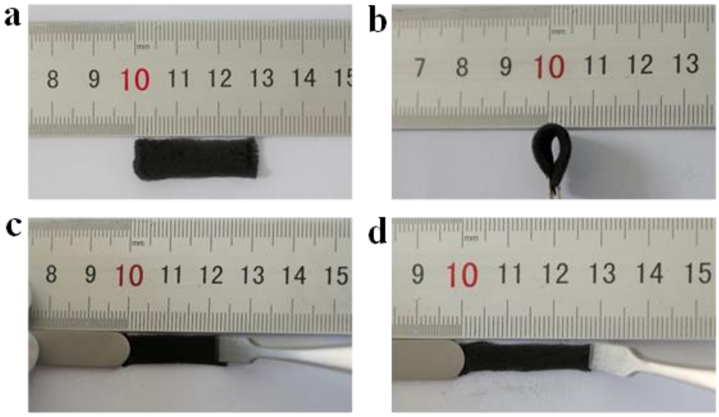

Figure S8. The (a and b) flexible and (c and d) tensile testing of NCNF@CS-6h.

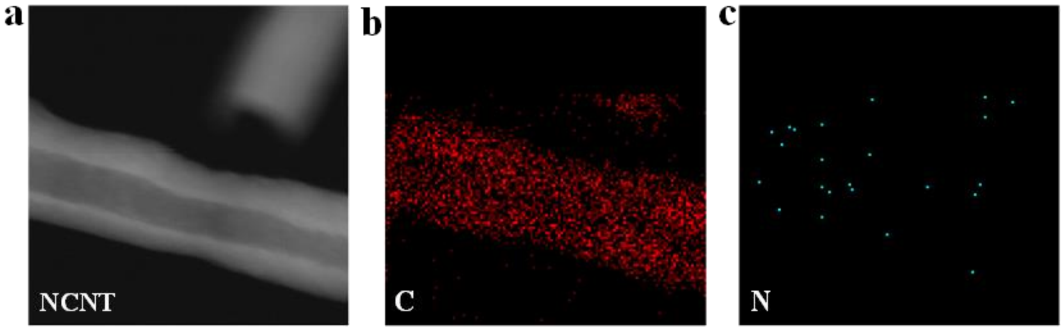

Figure S9. (a) HAADF-STEM image and EDS mappings of (b) C, and (c) N in NCNF.

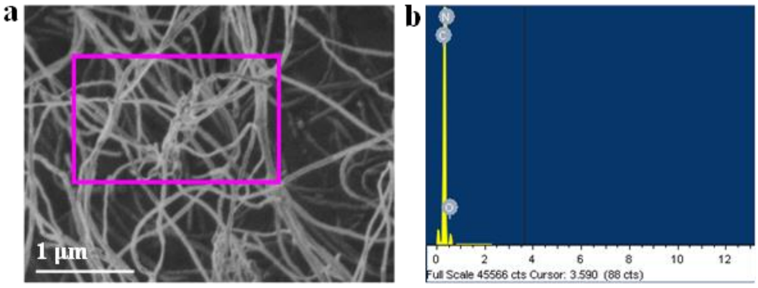

Figure S10. (a) SEM image and EDS spectra of NCNF.

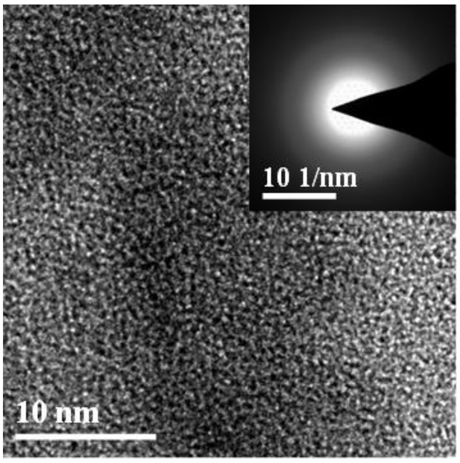

Figure S11. HRTEM image and the corresponding SAED pattern (inset) of CoO_x in NCNF@CoO_x-6h.

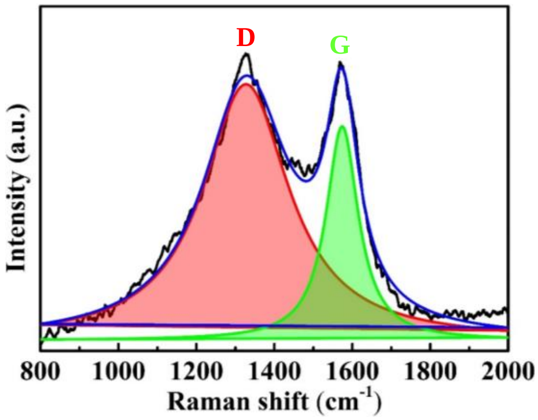

Figure S12. Raman spectrum of NCNF@CS-6h.

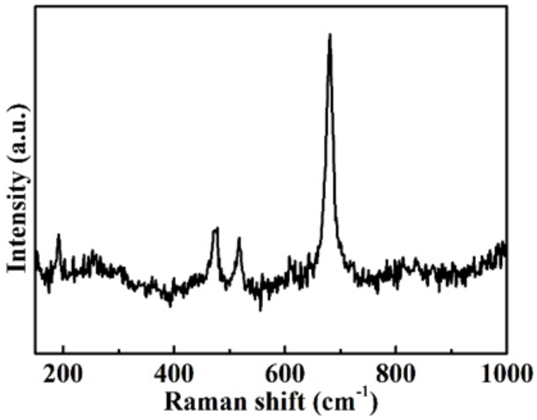

Figure S13. Raman spectra of cobalt selenide.

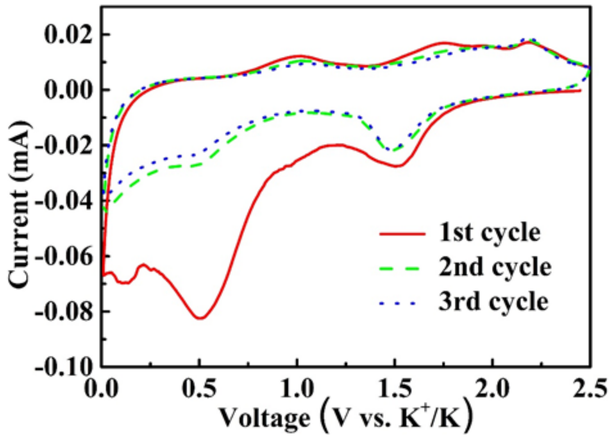

Figure S14. CV curves of NCNF@CS-6h for the initial 3 cycles at a sweep rate of 0.1 mV s^{-1}.

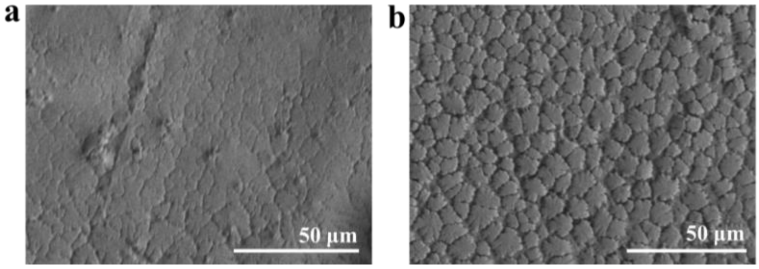

Figure S15. The SEM images of K metal surface after (a) 3 cycles and (b) 100 cycles.

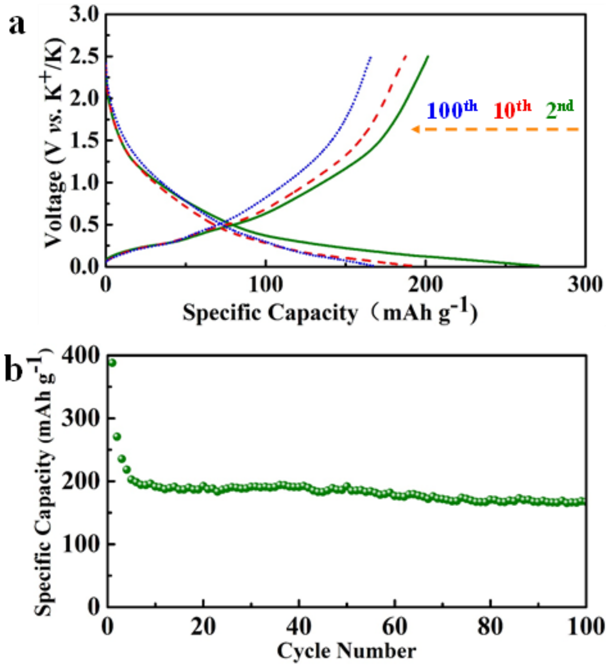

Figure S16. (a) The 2nd, 10th and 100th charge/discharge profiles of NCNF. (b) Cycling stability of NCNF at 0.2 A g^{-1} for KIBs.

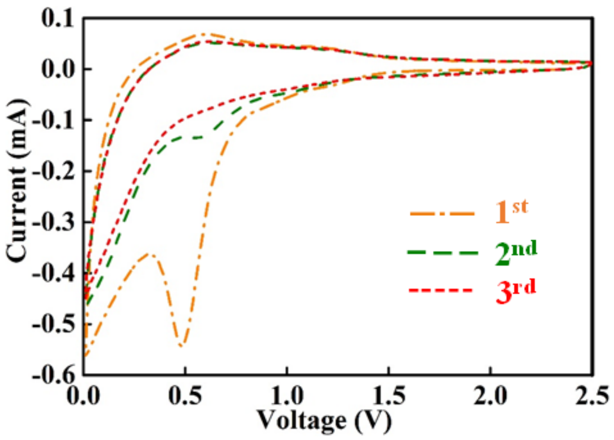

Figure S17. CV curves of NCNF for the initial 3 cycles at a sweep rate of 0.1 mV s^{-1}.

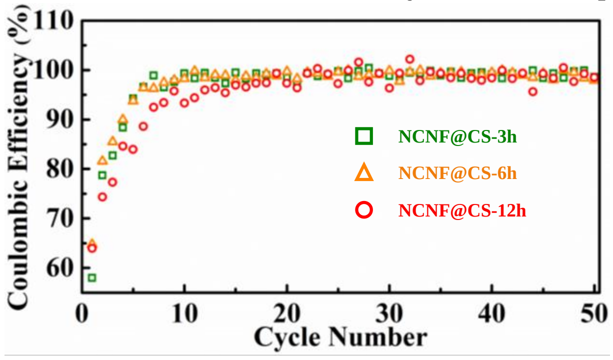

Figure S18. The corresponding Coulombic efficiencies of the as-prepared three samples.

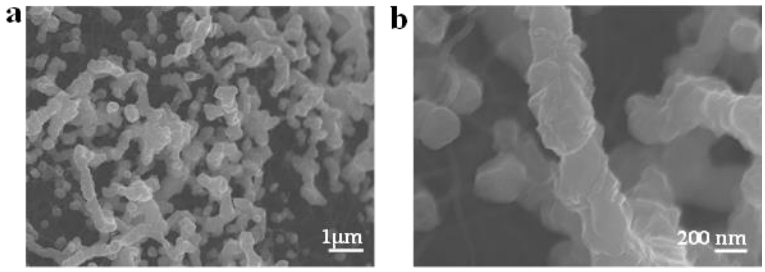

Figure S19. SEM images of NCNF@CS-6h after 600 cycles.

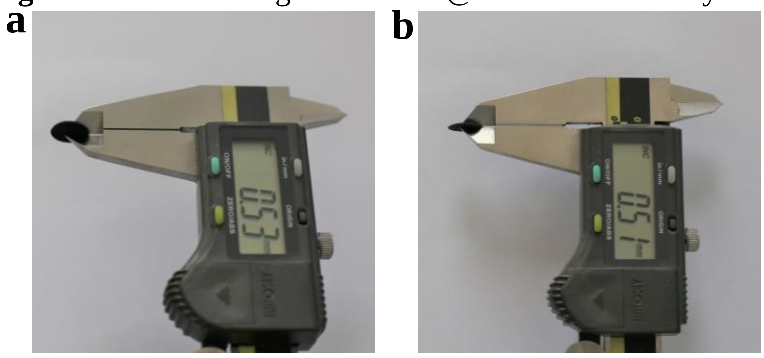

Figure S20. Optical photographs of the NCNF@CS-6h electrode: (a) original and (b) after 200 cycles.

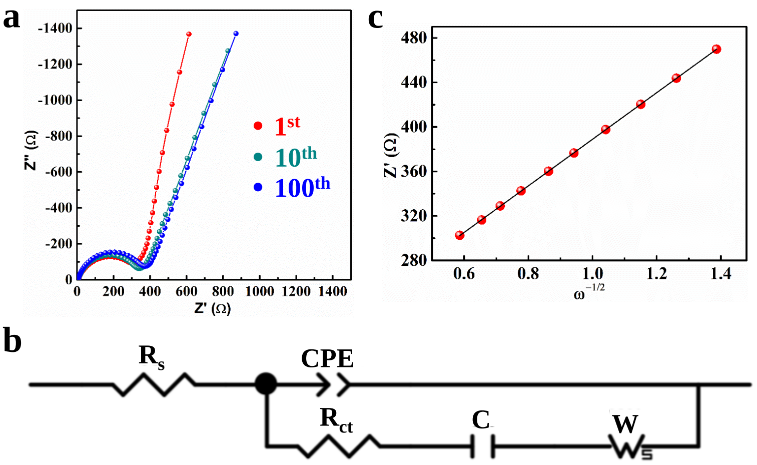

Figure S21. (a) nyquist plots, (b) the linear fits of the Z' *vs.* $\omega^{-1/2}$ in the low-frequency region and (c) the corresponding equivalent circuit of the coin cell.

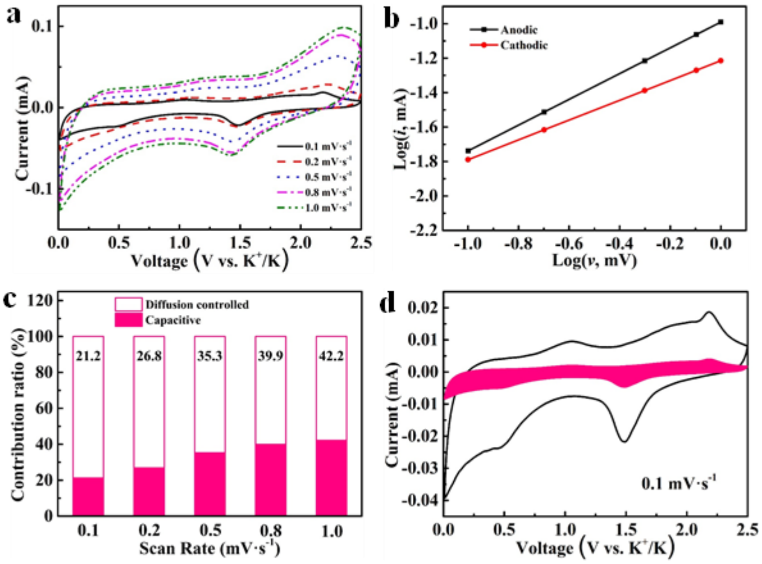

Figure S22. (a) CV profiles at different scan rates and (b) the plots of log(i) vs. log(υ) (peak current: i, scan rate: υ) of NCNF@CS-6h. (c) The percentages of pseudocapacitive contributions at different scan rates. (d) The red region shows the CV profile with the pseudocapacitive contribution at scan rate of 0.1 mV·s^{-1}.

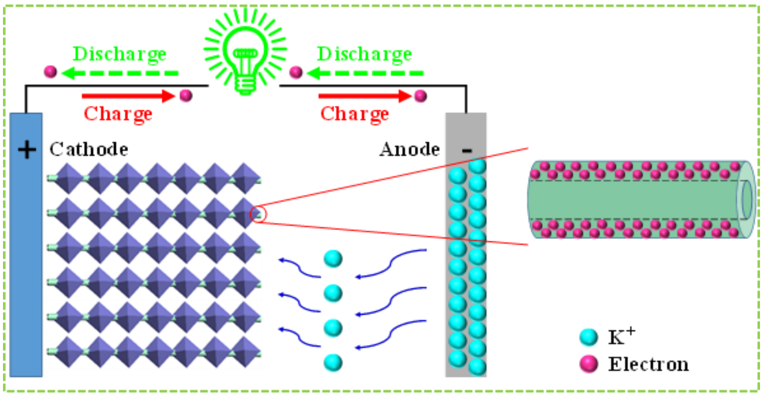

Figure S23. Schematic showing the electrochemical behavior in NCNF@CS host.

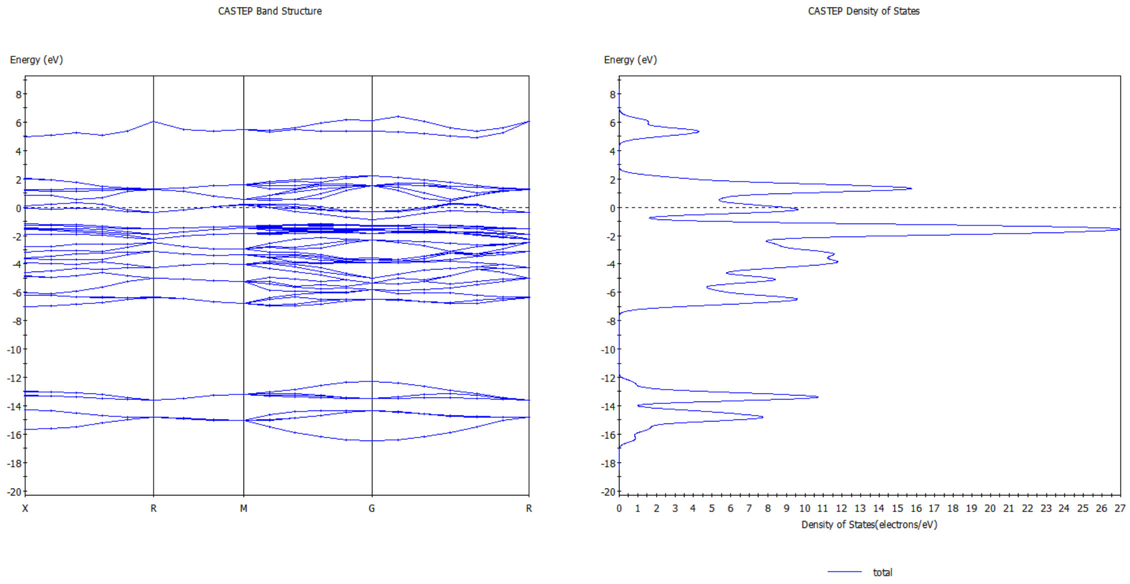

Figure S24. The bang gap structure and density of states of $CoSe_2$.

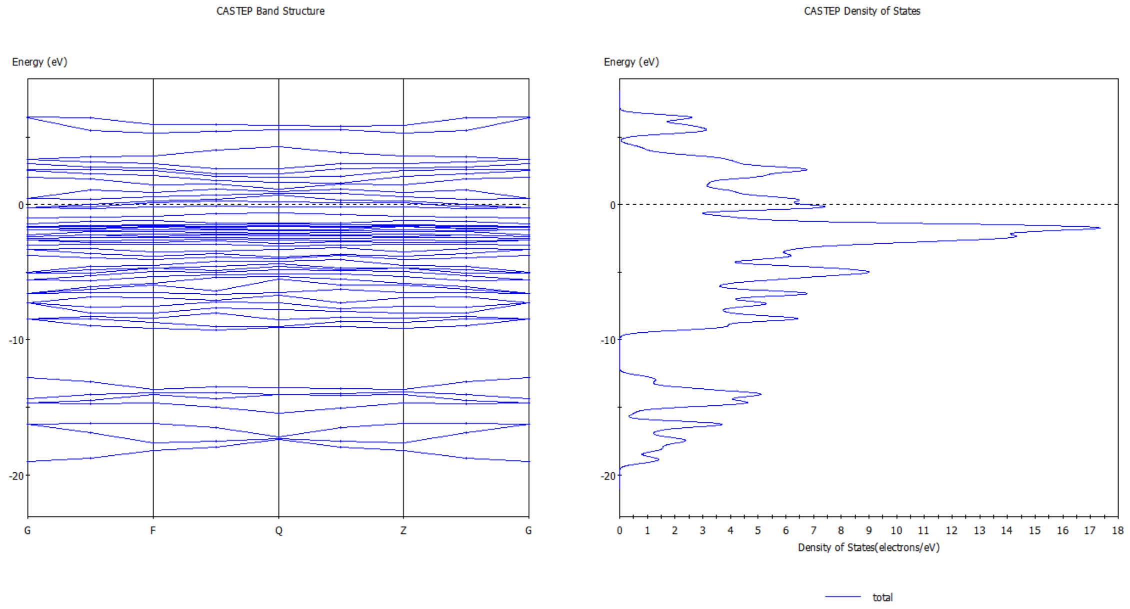

Figure S25. The bang gap structure and density of states of Co_3Se_4.

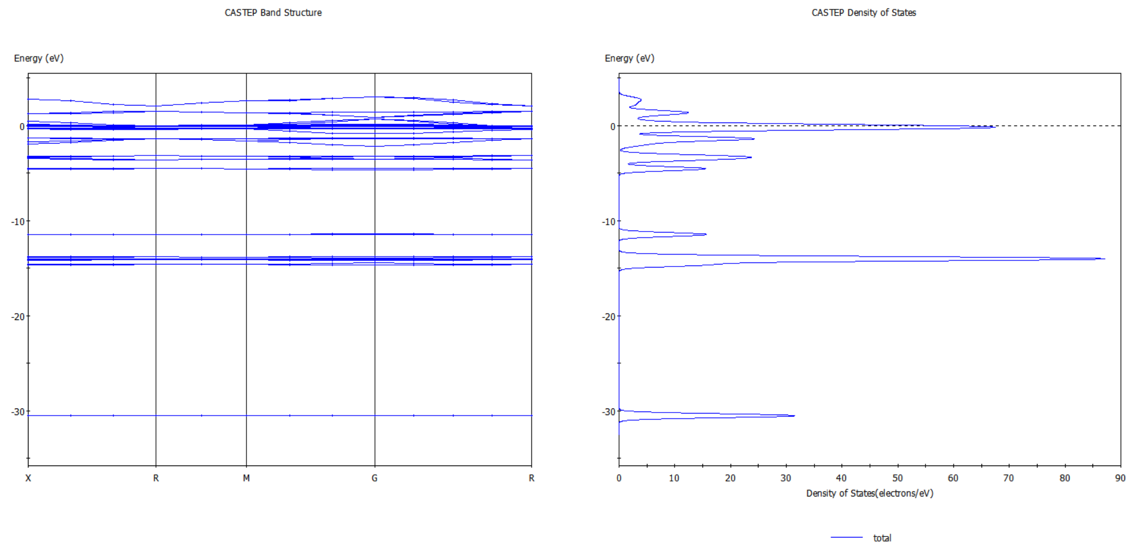

Figure S26. The bang gap structure and density of states of K_2CoSe_2.

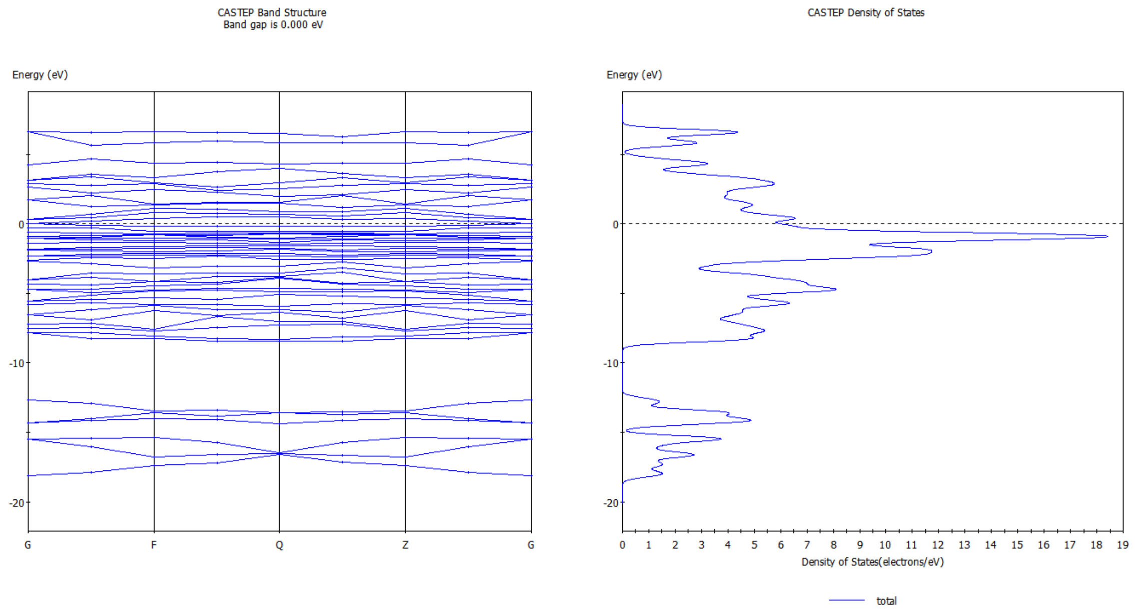

Figure S27. The bang gap structure and density of states of CoSe.

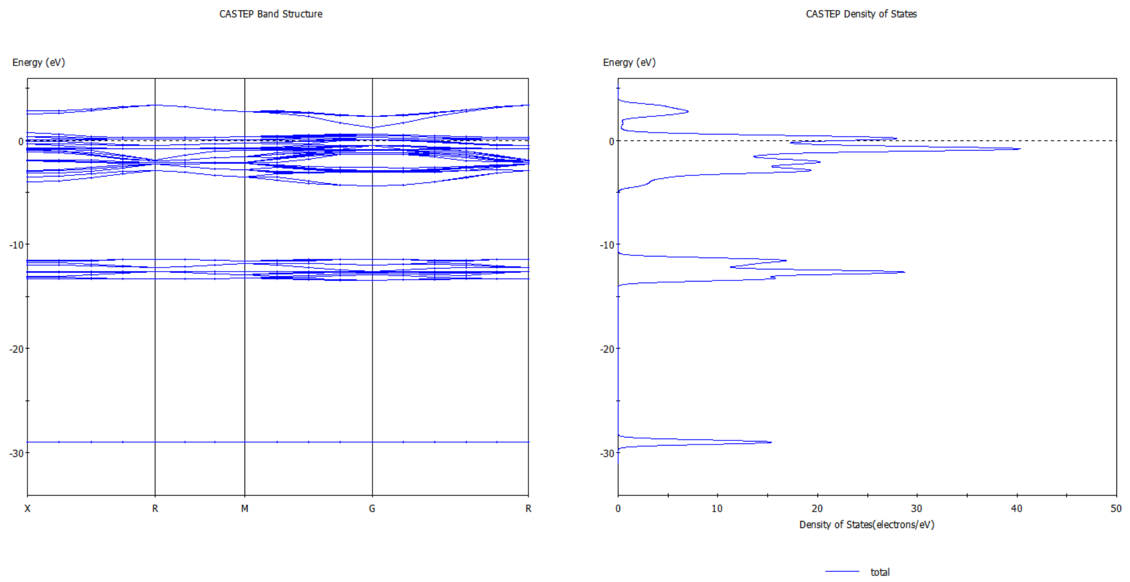

Figure S28. The bang gap structure and density of states of KCo_2Se_2.

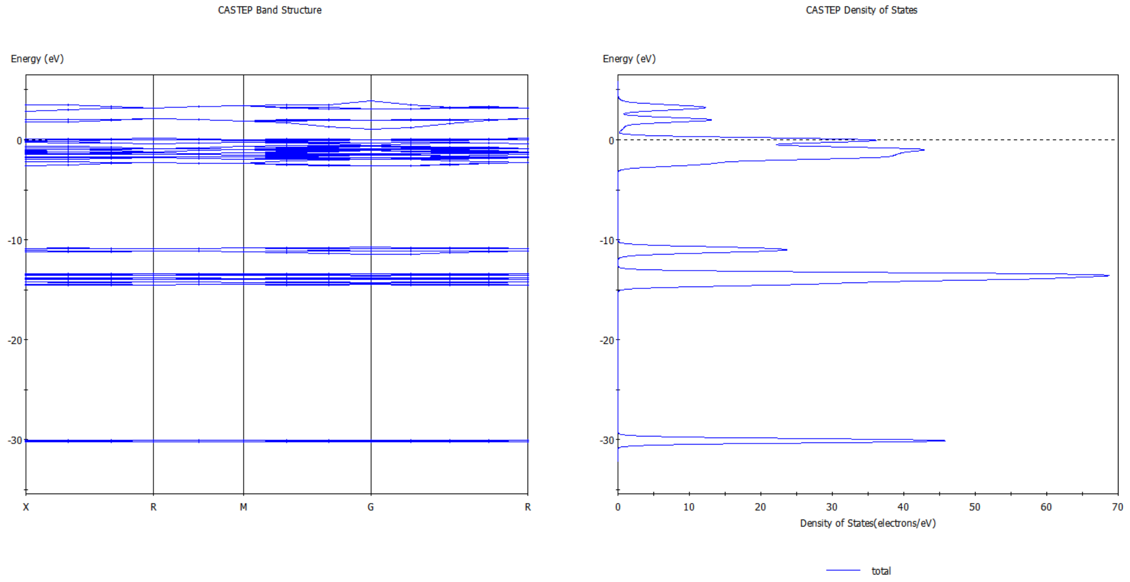

Figure S29. The bang gap structure and density of states of K_6CoSe_4.

Table S1. The weight percentage of N, C and H of NCNF@CS by elemental analyzer.

**Sample**	**Weight Percentage (%)**		
	N	C	H
**NCNF@CS-3h**	2.42	36.76	0.02
**NCNF@CS-6h**	2.28	27.32	0.05
**NCNF@CS-12h**	1.13	18.24	0

Table S2. Electrochemical performance comparison of the recent reported anode materials for KIBs.

Sample	Current density: A g^{-1}	Capacity: mAh g^{-1}	Cycle number	Reference
**NCNF@CS-6h**	**0.2**	**253**	**100**	**This work**
	**2**	**173**	**600**	
Graphite	0.14	100	50	[1]
Hard Carbon	0.028	216	100	[2]
N-doped Graphene	0.1	210	100	[3]
RGO Film	0.01	150	175	[4]
Graphite and Graphene	10	100	1000	[5]
Graphene	0.1	140	100	[6]
$K_2Ti_8O_{17}$	0.02	111	50	[7]
Sn/C	0.025	105	30	[8]
Dipotassium Terephthalate	0.2	229	100	[9]
3,4,9,10-perylene-tetracarboxylicacid-dianhydride	0.01	160	35	[10]
Sn_4P_3/C	0.05	307	50	[11]
Sb/C	0.035	250	50	[12]
Black P	0.05	270	50	[13]
SnS_2/rGO	0.025	250	30	[14]
Sb_2S_3/S,N-codoped rGO	0.05	477	100	[15]

Table S3. The impedance parameters of the NCNF@CS-6h-based coin cell.

R_s (Ω)	R_{ct} (Ω)	σ(Ω $s^{-0.5}$)	D (cm^2 s^{-1})

5.72	245	209.25	4.34×10^{-10}

Table S4. The structure information and total energy of different compositions.

Sample	supercell	Space Group	a	b	c	α	β	γ	Volume	total energy(eV)
**$CoSe_2$**	Co_4Se_8	Pa-3	5.733	5.733	5.733	90	90	90	188.44	-66.27
**Co_3Se_4**	Co_4Se_8	C2/m	11.548	3.515	6.054	90	119.27	90	214.35	-81.99
**K_2CoSe_2**	$K_8Co_4Se_8$	Ibam	6.862	12.924	6.029	90	90	90	534.60	-83.84
**CoSe**	Co_2Se_2	P63/mmc	3.552	3.552	5.100	90	90	120	55.73	-24.06
**KCo_2Se_2**	$K_2Co_4Se_4$	I4/mmm	3.711	3.711	13.513	90	90	90	186.06	-53.45
**K_6CoSe_4**	$K_{12}Co_2Se_8$	P63mc	10.008	10.008	7.805	90	90	120	677.02	-62.78
**Co**	Co_2	P63/mmc	2.416	2.416	3.919	90	90	120	19.82	-14.99
**K**	K_2	Im-3m	5.216	5.216	5.216	90	90	90	141.92	-2.24
**K_2Se**	K_8Se_4	Fm-3m	7.617	7.617	7.617	90	90	90	441.99	-37.52
**$Co(K_a)Se_2$**	$Co_4K_4Se_8$	Pa-3	7.584	7.584	7.584	90	90	90	436.23	-62.52
**$Co(K_b)_2Se_2$**	$Co_4K_8Se_8$	Pa-3	8.244	8.244	8.244	90	90	90	560.25	-61.53
**$Co(K_a)(K_b)_2Se_2$**	$Co_4K_{12}Se_8$	Pa-3	9.528	9.528	9.528	90	90	90	865.11	-62.78
**Co_3KSe_4**	$Co_6K_2Se_8$	C2/m	17.468	3.365	8.636	90	140.57	90	322.38	-86.54

Table S5. The structure and bind gap of different compositions.

**Sample**	**$CoSe_2$**	**Co_3Se_4**	**K_2CoSe_2**	**CoSe**	**KCo_2Se_2**	**K_6CoSe_4**	**Co**	**K_2Se**
**supercell**	Co_4Se_8	Co_4Se_8	$K_8Co_4Se_8$	Co_2Se_2	$K_2Co_4Se_4$	$K_{12}Co_2Se_8$	Co_2	K_8Se_4
**Space Group**	Pa-3	C2/m	Ibam	P63/mmc	I4/mmm	P63mc	P63/mmc	Fm-3m
**Band Gap/eV**	0	0	0	0	0	0	0	2.108

References

[1] Z. Jian, W. Luo, X. Ji, *J. Am. Chem. Soc.* **2015**, *137*, 11566.

[2] Z. Jian, Z. Xing, C. Bommier, Z. Li, X. Ji, *Adv. Energy Mater.* **2016**, *6*, 1501874.

[3] K. Share, A. P. Cohn, R. Carter, B. Rogers, C. L. Pint, *ACS Nano* **2016**, *10*, 9738.

[4] W. Luo, J. Wan, B. Ozdemir, W. Bao, Y. Chen, J. Dai, H. Lin, Y. Xu, F. Gu, V. Barone, L. Hu, *Nano Lett.* **2015**, *15*, 7671.

[5] A. P. Cohn, N. Muralidharan, R. Carter, K. Share, L. Oakes, C. L. Pint, *J. Mater. Chem. A* **2016**, *4*, 14954.

[6] K. Share, A. P. Cohn, R. Carter, C. Pint, *Nanoscale* **2016**, *8*, 16435.

[7] J. Han, M. Xu, Y. Niu, G. Li, M. Wang, Y. Zhang, M. Jia, C. Li, *Chem. Commun.* **2016**, *52*, 11274.

[8] I. Sultana, T. Ramireddy, M. M. Rahman, Y. Chen, A. M. Glushenkov, *Chem. Commun.* **2016**, *52*, 9279.

[9] K. Lei, F. Li, C. Mu, J. Wang, Q. Zhao, C. Chen, Jun Chen, *Energy Environ. Sci.* **2017**, *10*, 552.

[10] Y. Chen, W. Luo, M. Carter, L. Zhou, J. Dai, K. Fu, S. Lacey, T. Li, J. Wan, X. Han, Y. Bao, L. Hu. *Nano Energy* **2015**, *18*, 205.

[11] W. Zhang, J. Mao, S. Li, Z. Chen, Z. Guo. *J. Am. Chem. Soc.* **2017**, *139*, 3316.

[12] W. D. McCulloch, X. Ren, M. Yu, Z. Huang, Y. Wu, *ACS Appl. Mater. Interfaces* **2015**, *7*, 26158.

[13] I. Sultana, M. M. Rahman, T. Ramireddy, Y. Chen, A. M. Glushenkov, *J. Mater. Chem. A* **2017**, *5*, 23506.

[14] V. Lakshmi, Y. Chen, A. A. Mikhaylov, A. G. Medvedev, I. Sultana, M. M. Rahman, O. Lev, P. V. Prikhodchenko, A. M. Glushenkov, *Chem. Commun.* **2017**, *53*, 8272.

[15] Y. Lu, J. Chen, *Sci. China Chem.* **2017**, *60*, 1533.
